# Single-cell profiling of peripheral blood and muscle cells reveals inflammatory features of juvenile dermatomyositis

**DOI:** 10.3389/fcell.2023.1166017

**Published:** 2023-04-20

**Authors:** Xiangyuan Chen, Dongsheng Lian, Huasong Zeng

**Affiliations:** ^1^ Department of Pediatrics, The First Affiliated Hospital, Jinan University, Guangzhou, China; ^2^ Department of Allergy, Immunology and Rheumatology, Guangzhou Women and Children’s Medical Center, Guangdong Provincial Clinical Research Center for Child Health, Guangzhou Medical University, Guangzhou, China; ^3^ Department Institute of Pediatrics, Guangzhou Women and Children’s Medical Center, Guangdong Provincial Clinical Research Center for Child Health, Guangzhou Medical University, Guangzhou, China

**Keywords:** single-cell RNA sequencing, juvenile dermatomyositis, muscle, CYP4F3+ monocytes, type I interferon, peripheral blood

## Abstract

**Introduction:** Juvenile dermatomyositis (JDM) is a rare yet serious childhood systemic autoimmune condition that primarily causes skin rashes and inflammatory myopathy of the proximal muscles. Although the associated immune response involves the innate and adaptive arms, a detailed analysis of the pertinent immune cells remains to be performed. This study aims to investigate the dynamic changes of cell type, cell composition and transcriptional profiles in peripheral blood and muscle tissues, and in order to clarify the involvement of immune cells in the pathogenesis of JDM and provide a theoretical reference for JDM.

**Methods:** Single-cell RNA sequencing combined with bioinformatic analyses were used to investigate the dynamic changes in cell composition and transcriptional profiles.

**Results:** Analysis of 45,859 cells revealed nine and seven distinct cell subsets in the peripheral blood and muscle tissues respectively. IFITM2+ and CYP4F3+ monocytes were largely produced, and CD74^+^ smooth muscle cells (SMCs) and CCL19+ fibroblasts were identified as inflammatory-related cell subtypes in JDM patients, exhibiting patient-specific cell population heterogeneity.The dynamic gene expression patterns presented an enhanced type I interferon response in peripheral blood monocytes and T-cells, and SMCs and fibroblasts in muscle of untreated JDM patients. EGR1 and IRF7 may play central roles in the inflammation in both CD74^+^ SMCs and CCL19+ fibroblasts. Moreover, inflammatory-related monocytes could regulate T-cells, and the interaction between immune cells and SMCs or fibroblasts in muscle was enhanced under the inflammatory state.

**Conclusions:** Immune dysregulation is one of the key pathogenic factors of JDM, and type I interferon responses are significantly enhanced in peripheral blood Monos and T cells as well as SMCs and fibroblasts. EGR1 and IRF7 may play central roles in the inflammation and are considered as potential therapeutic targets for JDM.

## 1 Introduction

Juvenile dermatomyositis (JDM) is a rare, complex, immune-mediated disease characterized by inflammation of the proximal musculature and skin (Wedderburn and Rider, 2009). The primary clinical symptoms include muscle weakness and skin rashes; however, some children also experience a more severe disease course that affects other organs, resulting in physical impairment, calcinosis, gastrointestinal perforations, interstitial lung disease, and even death (Mathiesen et al., 2012). Although the etiology is not fully understood, both environmental factors and genetic variations are thought to play a role (Miller et al., 2018). Although the use of corticosteroids has effectively reduced the mortality rate from >30% to 2%–3%, a long-term follow-up study found that >60% of children with JDM experience disease damage due to poor disease control or corticosteroid toxicity (Mathiesen et al., 2012). Thus, to improve patient outcomes, it is necessary to identify reliable and specific biomarkers capable of monitoring disease activity, predicting illness onset, and directing therapeutic regimens.

With the development of high-throughput technologies, including microarrays, bulk RNA sequencing (RNA-seq), and single-cell RNA-seq (scRNA-seq), progresses have been made toward understanding the pathogenesis of JDM. More specifically, several gene expression studies have reported dysregulation of genes in the muscles, skin, and peripheral blood of adult patients with dermatomyositis and JDM (Greenberg et al., 2005; Wenzel et al., 2006; Baechler et al., 2007; Neely et al., 2022; Stingl et al., 2022). Additionally, many proteins in the plasma, serum, and urine, as well as circulating immune cell subsets, have been investigated as potential biomarkers for JDM (Wienke et al., 2018). However, the immune mechanisms of JDM are complex, involving the innate and adaptive arms. Recent studies have revealed immune dysregulation in innate, humoral, and cellular responses in patients with JDM (Elst et al., 2008; O'Connor et al., 2006; Wedderburn et al., 2007). Moreover, the inflammatory process in JDM is characterized by interferon (IFN) features and infiltration of specific immune cell subsets, including T-cells and plasmacytoid dendritic cells (DCs) (Wienke et al., 2018). However, to date, it remains unclear which immune cells contribute to the development and pathogenesis of this disease.

In this study, we sought to provide an in-depth characterization of cell types in the peripheral blood and muscle tissues of patients with JDM, as well as insights into the dynamic changes in cell composition and transcriptional profiles associated with JDM pathogenesis. scRNA-seq and transcriptomic analyses assessed the composition of the major cell types, including T-cells, natural killer T-cells (NKT), natural killer cells (NK), classical-monocytes, non-classical-monocytes, dendritic cells (DCs), B-cells, plasma cells, and platelets, within the peripheral blood, and T-cells, NK, B-cells, non-classical-monocytes, endothelial cells, fibroblasts, and smooth muscle cells (SMC) within the muscle tissues. Moreover, we defined the inflammatory cell subtypes of monocytes and T-cells in the peripheral blood and SMCs and fibroblasts in muscles. Collectively, our data provide a comprehensive resource regarding JDM pathogenesis, which will inform the development of effective therapeutic strategies.

## 2 Materials and methods

### 2.1 Clinical sample collection and cell dissociation

Four patients meeting the Bohan and Peter criteria for JDM were admitted to the Guangzhou Women and Children’s Medical Center in China and enrolled in this study from February to December 2020, with an average age of 7.6 years old at the time of sampling (Bohan and Peter, 1975). The detail informations of the four cases can be gained at Supplementary Material S1. Informed written consents were obtained from participants and their parents. JDM patients in the pre-treatment group met the diagnostic criteria of Bohen and Peter without having received any previous or current drug treatment; JDM patients in the post-treatment group met the diagnostic criteria of Bohen and Peter and were treated with prednisone, hydroxychloroquine, and cyclophosphamide for 3–6 months according to the JDM treatment guidelines.

Peripheral blood and muscle tissue samples for scRNA-seq were typically collected at the time of admission or discharge. Peripheral blood samples were collected from two pre- and two post-treatment JDM patients, mixed with ethylenediaminetetraacetic acid (EDTA) (Sangon Biotech, Shanghai, China) solution in a 1:1 volume, transferred to a 50 mL centrifuge tube, and centrifuged at 400 × g for 4 min twice. Blood cells were resuspended in Dulbecco’s phosphate-buffered saline (DPBS) (Sangon Biotech, Shanghai, China) containing 0.04% bovine serum albumin (BSA) (Rockland, Philadelphia, United States).

Muscle tissues were collected from only the two pre-treatment JDM patients as the post-treatment patients’ muscle strength returned to normal, making it unnecessary to conduct an invasive muscle biopsy (the parents/guardians also refused consent). Fresh muscle tissues were washed with DPBS and cut into small pieces. Muscle cells were dissociated using a combination of enzymatic digestion with 0.2 mg/mL Dispase (Corning, New York, United States), 2 mg/mL type II collagenase (Gibco, New York, United States), 2 mg/mL type IV collagenase (Gibco, New York, United States), and 12 UI/mL DNAase (Macklin, Shanghai, China) at 37°C for 20 min. The cell-enzyme mixture was passed through a 40-µM stainless nylon mesh (Greiner Bio-OneGmbH, Germany), and the filtrate was centrifuged at 500 × g for 5 min. The cell sediment was resuspended and lysed with red blood cell (RBC) Lysis buffer (Boster, California, United States)on ice for 5 min to remove red blood cells, and then washed twice with DPBS at 500 × g for 5 min. The cells were resuspended in DPBS containing 0.04% BSA for single-cell capture.

Cell concentration and viability were determined using a Cellometer Auto 2000 (Nexcelom, Boston, United States) following acridine orange propidium iodide (AO/PI) (Macklin, Shanghai, China) staining. Cells with >80% viability were subjected to 10× Genomics scRNA-seq.

This study was approved by the hospital ethics committee (approval number: 2020-32401) of Guangzhou Women and Children’s Medical Center. All experiments and samples were performed in accordance with the ethical and biosafety protocols approved by the institutional guidelines. The clinical trial registration number is ChiCTR2000034590.

### 2.2 cDNA library preparation and single-cell RNA sequencing data processing

Peripheral blood cells from pre- and post-JDM patients and muscle cells from JDM patients were loaded into a Chromium Controller (10× Genomics, California, United States) according to the standard protocol to capture cells. cDNA synthesis and library preparation were performed following the manufacturer’s instructions of the Single Cell 3′Reagent Kits User Guide Version 3.1 (10× Genomics, California, United States), and subsequently subjected to high-throughput sequencing on an Illumina NovaSeq 6,000 using paired-end 150 bp sequencing runs.

For comparison, publicly available data for normal tissues were selected as the control groups. The blood cell dataset for the control was obtained from a healthy donor, downloaded from the 10× Genomics website (https://www.10xgenomics.com/resources/datasets/10-k-pbm-cs-from-a-healthy-donor-v-3-chemistry-3-standard-3-0-0); muscle cell datasets for healthy controls were obtained from the Gene Expression Omnibus (GEO; https://www.ncbi.nlm.nih.gov/geo/) with the accession codes GSM6611295 and GSM6611297. The raw data for the peripheral blood cells of pre- and post-treatment JDM patients and JDM muscles are publicly deposited at the National Genomics Data Center (https://ngdc.cncb.ac.cn/gsa-human, the accession number is HRA004355).

CellRanger (version 3.1.0) was used to align raw reads on the GRCh38 reference genome for humans and to generate unique molecular identifier (UMI) gene expression profiles for each single cell under the standard sequencing quality threshold (default parameters). High-quality cells were retained for downstream analysis when they met the following criteria: 1) peripheral blood cells with >500 and <5,000 unique genes, and muscle cells with >100 and <6,000 unique genes; 2) less than 10% mitochondrial genome transcripts (peripheral blood cells) or no mitochondrial genes (muscle cells). Doublets in the raw data were identified and removed using DoubletFinder (https://github.com/chris-mcginnis-ucsf/DoubletFinder) within a range of 7.5%. Ultimately, 45,859 cells and 30,786 genes were deemed to be of sufficiently high quality for further analysis.

### 2.3 Data normalization and cell clustering

The final filtered gene expression data matrix was normalized using the NormalizeData function of the Seurat software (https://satijalab.org/seurat/pbmc3k_tutorial.html) with default settings. We selected 3,000 highly variable genes *via* the FindVariableGenes function from the final filtered count matrix and then centered and scaled them using ScaleData. To reduce the dimensionality of the datasets, principal component analysis (PCA) on the 3,000 genes was conducted using the RunPCA function with default parameters, and the dimensional reduction was performed through a Canonical correlation analysis (CCA) method. Cells were clustered using the FindNeighbors and FindClusters functions by setting the clustering parameter resolution to 0.4 (peripheral blood cell) or 0.2 (muscle cell). The clustered cells were then projected onto a two-dimensional space using Uniform Manifold Approximation and Projection (UMAP) (https://satijalab.org/seurat/articles/get_started.html) with dimension parameters of 1:30 (peripheral blood cell) or 1:50 (muscle cell) for visualization.

### 2.4 Cell-type subclustering

Several critical cell types were sub-clustered to further explore their heterogeneity and functional changes in patients with JDM. The same functions described above were used to obtain the sub-clusters. Briefly, the main cell cluster was identified using the Louvain-Jaccard graph-based method following dimensionality reduction by PCA. The clustering parameter resolution was set to 0.3 for the FindClusters function, while the RunUMAP function dimension parameter was set to 1:30 (peripheral blood cell) or 1:50 (muscle cell). Seurat was used to achieve dimensional reduction for visualization.

### 2.5 Differential proportion analysis

Differential proportion analysis was performed according to changes in the cell ratio under different conditions. First, we determined the proportion of each cell type or subtype by dividing the number of cells by the total number of cells in the different groups. Next, the Log2-fold change (log2FC) was calculated between the healthy control and pre-treatment samples and between the pre-treatment and post-treatment samples; |log2FC|> 0.5 was set as the threshold for significant change.

### 2.6 Differentially expressed gene analysis and gene-enrichment analysis

Differentially expressed genes (DEGs) between pre-treatment and healthy control groups of peripheral blood and muscle, and between post-treatment and pre-treatment groups of peripheral blood were identified by the FindMarkers function in Seurat using the Wilcoxon rank sum test with Bonferroni correction. Significant DEGs were selected from genes with an adjusted *p*-value ≤0.05, |log2FC| ≥ 0.5, and those expressed in >10% of the cells within either or both groups. Gene Ontology (GO) enrichment analysis of these significant DEGs was performed using the clusterProfiler (v3.14.3) R package (Yu et al., 2012). Enrichment scores (*p*-values) for selected numbers of GO annotations were calculated using a hyper-geometrical statistical test with a threshold of 0.05, and the Benjamini–Hochberg method was used to estimate the false discovery rate (FDR). The background for human data comprised all genes listed in the org. Hs. e.g., db database. Finally, the bar plot function was used for visualization.

### 2.7 Pseudotime trajectory construction

The gene expression profiling data of all single cells was applied to deconstruct population heterogeneity and reprogram trajectory. The cells were ordered in a pseudo-temporal manner using the Monocle 2 R package (http://cole-trapnell-lab.github.io/monocle-release/monocle2/). The count data and metadata were exported from the Seurat object and imported into the CellDataSet object in Monocle 2. The raw counts for cells in each cell type were extracted and normalized with the estimateSizeFactors and estimateDispersions functions with the default parameters. Only genes matching the following thresholds were used for cell ordering and training the pseudo-trajectory: 1) mean expression >0.1; 2) dispersion_empirical >1.2*dispersion_fit. The orders were determined by the orderCells function, while the trajectory was inferred by the reduceDimension function with default parameters. Finally, the reconstructed trajectories were visualized using the plot_cell_trajectory function.

### 2.8 Cellular interaction analysis

CellPhoneDB (2.1.2) (https://www.cellphonedb.org/), a publicly available repository of curated receptors, ligands, and their interactions (Vento-Tormo et al., 2018), was used to investigate the potential interactions between different cell types. The normalized counts and cell subset annotations for each cell were inputted into CellPhoneDB to determine potential ligand-receptor pairs. The communication probability of ligand-receptor pairs was determined according to the mass action law of the average expression of a ligand by 1 cell population and the average expression of a receptor by another cell population. The significant ligand-receptor pairs were filtered based on *p*-values <0.05, and average expression of interaction pairs >0. The interaction networks of selected specific pairs were plotted using the dot_plot function of CellPhoneDB with default parameters.

## 3 Results

### 3.1 scRNA-seq maps distinct cell populations in peripheral blood cells and muscle cells from control and JDM patients

**FIGURE 1:**
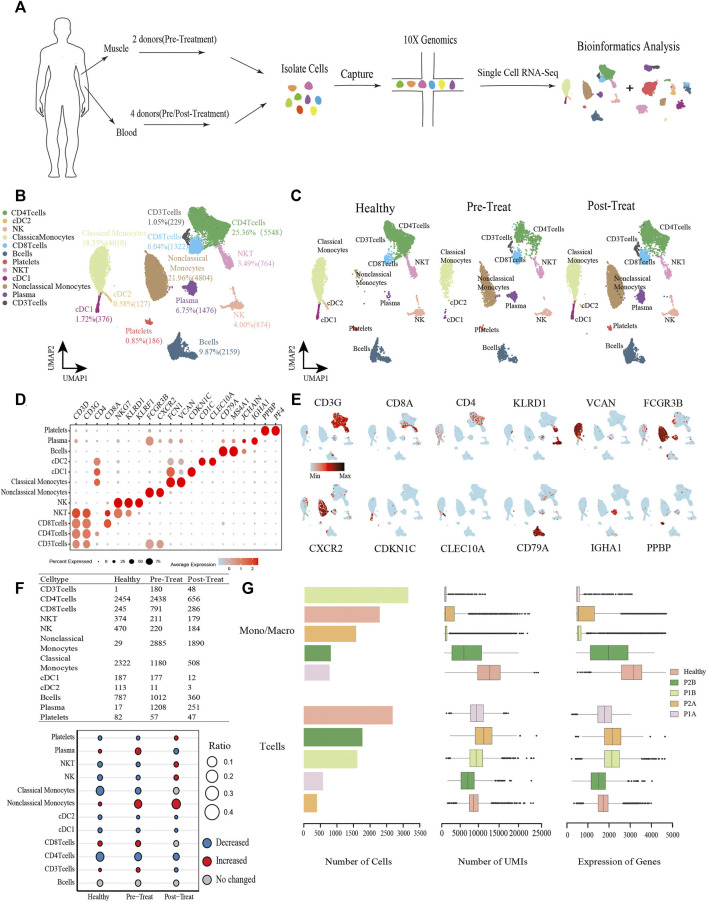
Global transcriptomic profiles of peripheral blood cells from control and JDM patients reveals their cellular subpopulations as determined by ScRNA-seq. **(A)** Overall workflow for cell sorting and single-cell data analyses. **(B)** Uniform Manifold Approximation and Projection (UMAP) plots of single-cell transcriptomic profiles showing cell types of peripheral blood cells from all the samples. Each dot represents a cell, which is colored according to cell type. **(C)** UMAP plots of single-cell transcriptomic profiles showing cell types of peripheral blood cells from healthy, pre-treatment, and post-treatment groups, respectively. Each dot represents a cell, which is colored according to cell type. **(D)** Bubble chart of markers for each identified cell type. **(E)** Feature plots of expression distribution for representative genes for each cell type in peripheral blood. Expression levels for each cell are color-coded and overlaid into UMAP plot. **(F)** Heterogeneity of cell type composition in healthy control, pre-treatment, and post-treatment groups. The table shows the number of each cell type in each group (up); and the bubble chart represents the changes in the cell ratio of cell types between the pre-treatment and healthy control groups, and between the post- and pre-treatment groups in peripheral blood (down). **(G)** The number of cells, and box plots of the number of UMIs and genes (with the box plot center, box, whiskers, and points corresponding to the median, interquartile range, 1.5× interquartile range, and outliers, respectively) of Monos (a conbined cell type of monocytes and macrophages) and T cells in each sample of peripheral blood. Healthy was the sample from control group, P1B and P2B, and P1A and P2A were samples from pre-treatment group and post-treatment group respectively.

To understand the effects of JDM on the cell subset composition and transcriptional profiles of peripheral blood and muscle tissues in children, the peripheral blood cells from two pre-treatment and two post-treatment patients with JDM and muscle tissues from two pre-treatment patients were collected to prepare cell suspensions for scRNA-seq; data from healthy controls were included for further bioinformatics analysis to explore the molecular mechanisms (Figure 1A). From the peripheral blood sample, a total of 14,794 cells were isolated from all patient samples, with an average of 1,480 detected genes per cell. The dataset for the control sample comprised 7,081 cells with an average of 2,287 detected genes per cell. Following gene expression normalization, we applied PCA using the top variable genes ranked by their normalized dispersion, as previously described (Zheng et al., 2017). Unsupervised clustering using the Seurat package identified nine distinct cell types in peripheral blood cells across all three groups (healthy control, pre-treatment, and post-treatment groups), including three T-cell subsets (CD3^+^CD4^−^CD8^−^ T-cells, 1.05%; CD4^+^ T-cells, 25.36%; and CD8^+^ T-cells, 6.04%), NKT-cells (3.49%), NK cells (4.00%), non-classical-monocytes (21.96%), classical-monocytes (18.33%), two subtypes of DCs (cDC1, 1.72%; cDC2, 0.58%), B-cells (9.87%), plasma cells (6.75%), and platelets (0.85%) (Figures 1B, C), each identified by their unique signature genes (Figures 1D, E, Supplementary Table S1).

When comparing the proportions of cell populations among the three groups, we found that the percentages of plasma cells, CD3^+^ T-cells, CD8^+^ T-cells, and non-classical-monocytes were first significantly increased (|log2-FC|> 0.5) in pre-treatment group relative to control, and then plasma cells and CD3^+^ T-cells recovered to control levels in post-treatment group, while non-classical-monocytes continue to increase (|log2-FC|> 0.5) and CD8^+^ T-cells maintained stable levels in the post-treatment group compared with the pre-treatment group (Figure 1F). Whereas, platelets, NKT-cells, NK cells, classical-monocytes, cDC1, cDC2, and CD4^+^ T-cells were first significantly decreased (|log2-FC|> 0.5) in the pre-treatment group relative to the control group, and then platelets, NKT-cells, NK cells recovered to control levels in post-treatment group, while cDC1, cDC2, and CD4^+^ T-cells continue to decrease (|log2-FC|> 0.5) and classical-monocytes maintained stable levels in the post-treatment group compared with the pre-treatment group (Figure 1F). Notably, a significant decrease was observed in the classical-monocyte proportion between the pre-treatment and control groups (from 32.79% in control to 11.38% in the pre-treatment group) and a significant increase in that of non-classical-monocytes (from 0.41% in control to 27.82% in the pre-treatment group). Moreover, proportions of CD3^+^ T-cells and CD8^+^ T-cells were significantly increased in the pre-treatment group relative to the control (from 0.01% to 1.74%, and from 3.46% to 7.63%, respectively) (Figure 1F). The number of cells, UMIs and genes of monocytes (Monos, conbined classical-monocytes and non-classical-monocytes) and T-cells in each sample of peripheral blood were shown in Figure 1G.

**FIGURE 2:**
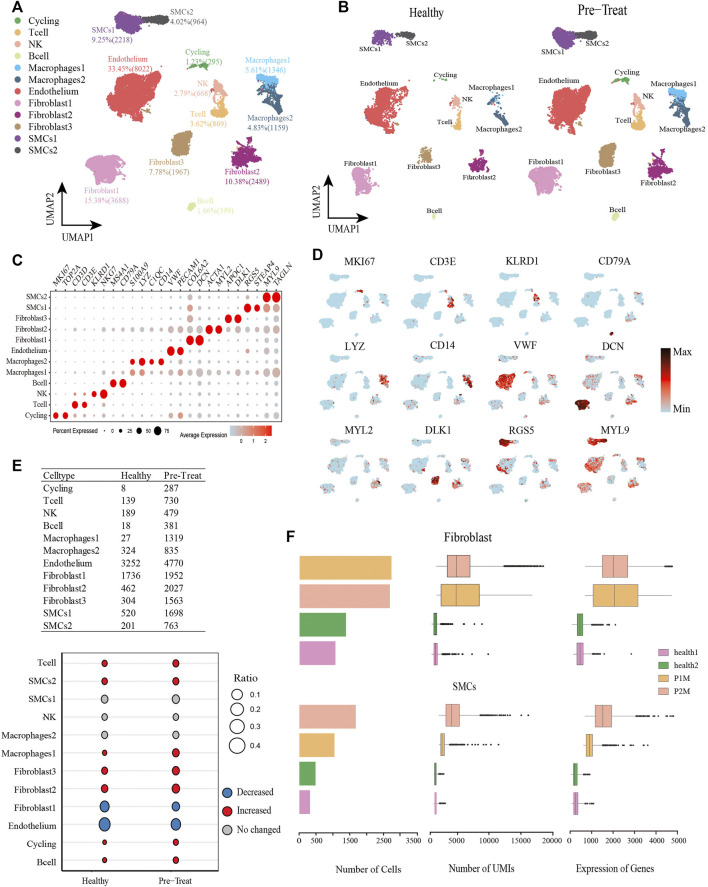
Global transcriptomic profiles of muscle cells from control and JDM patients reveals their cellular subpopulations as determined by ScRNA-seq. **(A)** Uniform Manifold Approximation and Projection (UMAP) plots of single-cell transcriptomic profiles showing cell types of muscle cells from all the samples. Each dot represents a cell, which is colored according to cell type. **(B)** UMAP plots of single-cell transcriptomic profiles showing cell types of muscle cells from healthy and pre-treatment groups, respectively. Each dot represents a cell, which is colored according to cell type. **(C)** Bubble chart of markers for each identified cell type. **(D)** Feature plots of expression distribution for representative genes for each cell type in muscle. Expression levels for each cell are color-coded and overlaid into UMAP plot. **(E)** Heterogeneity of cell type composition in healthy control and groups. The table shows the number of each cell type in each group (up); and the bubble chart represents the changes in the cell ratio of cell types between the pre-treatment and healthy control groups in muscle (down). **(F)** The number of cells, and box plots of the number of UMIs and genes (with the box plot center, box, whiskers, and points corresponding to the median, interquartile range, 1.5× interquartile range, and outliers, respectively) of SMCs and fibroblasts in each sample of muscle.

From the muscle tissue samples, 16,804 qualified cells were obtained from two pre-treatment JDM patients, with an average of 2,018 genes detected per cell. The datasets of muscle cells for healthy controls comprised 7,180 cells with an average of 609 genes detected per cell. We detected seven distinct cell types in the muscle cells of healthy control and pre-treatment groups: T-cells (3.62%), NK cells (2.79%), B-cells (1.66%), two subtypes of macrophages (macrophage1, 5.61%; macrophage2, 4.83%), endothelial cells (33.45%), three subtypes of fibroblasts (fibroblast1, 15.38%; fibroblast2, 10.38%; fibroblast3, 7.78%), two subtypes of SMCs (SMC1, 9.25%; SMC2, 4.02%), and a proliferating cell population designated ‘cycling’ (1.23%) (Figures 2A, B), which were annotated based on their signature genes (Figure 2C, Supplementary Table S2). Representative genes for each cell subtype are shown in the feature plots (Figure 2D). Among these muscle cells, we found that the proportions of T-cells, SMC2, macrophage1, fibroblast2, fibroblast3, cycling, and B-cells were significantly increased (|log2-FC|> 0.5), whereas fibroblast1 and endothelial cells were significantly decreased (|log2-FC|> 0.5) in JDM patients (pre-treatment group) compared to that in healthy controls (Figure 2E). Moreover, the number of cells, UMIs, and genes related to fibroblasts and SMCs in each muscle tissue are shown in Figure 2F.

### 3.2 Functional changes within the peripheral blood monocyte subsets and their pseudo-time trajectory reconstruction

**FIGURE 3:**
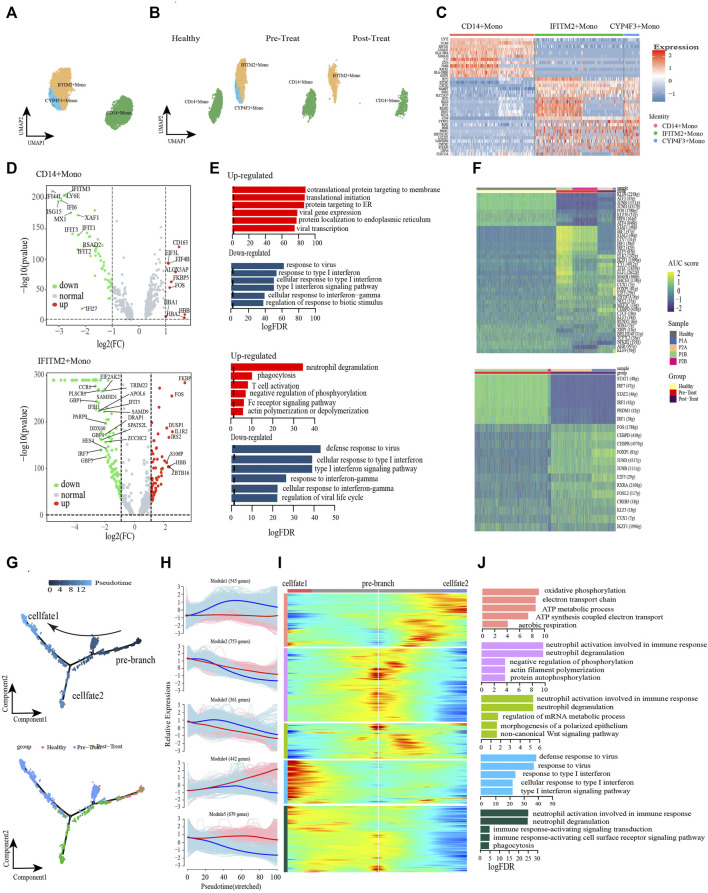
Classical monocytes were differentiated into non-classical monocytes in circulation of JDM patients. **(A, B)** Visualization of monocyte sub-clusters in human peripheral blood sample *via* Uniform Manifold Approximation and Projection (UMAP) plots by cell identity **(A)** and different stages **(B)**. **(C)** A heatmap shows the top marker genes of monocyte sub-clusters. **(D)** Volcano plot illustrating the representative differential genes between post-treat group and pre-treat group of CD14^+^ monocyte (up) and IFITM2+ monocyte (down). **(E)** GO enrichment of upregulated (red) and downregulated (blue) DEGs of CD14^+^ monocyte (up) and IFITM2+ monocyte (down) in the post-treatment group compared to pre-treat group. **(F)** CD14^+^ monocyte (up) and IFITM2+ monocyte (down) subpopulation-specific regulons identified *via* SCENIC analysis. **(G)** Pseudotime trajectory (Monocle analysis) of the monocyte. Cells are colored based according to the predicted pseudotime (top) and groups (bottom). **(H)** The expression dynamics of top DEGs were cataloged into five major clusters in a pseudotime manner shown as red lines (cell fate 1) and blue lines (cell fate 2). **(I)** A heatmap shows the different expression patterns of top DEGs (cataloged in five clusters) along the development of Cell fate1 and cell fate 2. **(J)** GO enrichment analyses of each gene cluster.

We further turned our attention to the monocyte populations because of the significant compositional changes observed between pre-treated JDM patients and healthy control, and explored the heterogeneity and functional changes within combined classical-monocytes and non-classical-monocytes (hereafter designated Monos) of the peripheral blood during the pathogenic process of JDM. Based on the expression levels of the highly enriched cluster markers identified in this study, we assigned the subsets of these Monos into three subclusters: CD14^+^ Monos (VCAN^high^ and CD14^high^), IFITM2+ Monos (IFITM2^high^ and CD16^high^), and CYP4F3+ Monos (CYP4F3^high^ and CD16^high^) (Figures 3A–C, Supplementary Table S3). Only CD14^+^ Monos were found in healthy controls, while all three subtypes were detected in the pre-treatment group, and CYP4F3+ Monos were absent in post-treatement patients (Figure 3B).

To further investigate the transcriptomic changes of Monos in peripheral blood during the pathogenic process of JDM, the expression patterns of the Monos subpopulations were compared between pre-treatment and post-treatment conditions. A total of 621 DEGs were identified in CD14^+^ Monos between the two groups, including 332 upregulated (e.g., *HBB*, *CD163*, *ALOX5AP*, *FKBP5*, *FOS*) and 289 downregulated genes (e.g., *IFI44L*, *ISG15*, *IFI6*, *IFITM3*, *LY6E*) in the post-treatment group relative to the pre-treatment group (Figure 3D up). GO enrichment analyses of these DEGs revealed enrichment of the upregulated genes in protein localization to the membrane and mRNA catabolic and translation processes, whereas the response to viruses and response to type I IFN were the most significantly downregulated pathways in CD14^+^ Monos (Figure 3E up). Subsequently, a heatmap revealed differentially expressed transcription factors (TFs), including KFL6, STAT1, ELK3, among the three CD14^+^ Monos populations (healthy control, pre-treatment, and post-treatment groups; Figure 3F).

Meanwhile, 881 DEGs were found in IFITM2+ Monos between the pre-treatment and post-treatment groups, comprising 519 upregulated (e.g., *FKBP5*, *IL1R2*, *FOS*, *ZBTB16*, *IRS2*) and 362 downregulated (e.g., *IRF7*, *TRIM22*, *DDX60*, *IFIT5*, *HES4*) DEGs in the post-treatment group (Figure 3D down). GO enrichment results indicated that processes associated with the immune defense response and RNA metabolic processes, e.g., phagocytosis, T-cell activation, and mRNA catabolic processes, were significantly upregulated in IFITM2+ Monos (Figure 3E down). In contrast, functional processes associated with the response to IFN/viruses (defense response to virus, response to virus, response to type I IFN, response to IFN-γ, *etc.*) in IFITM2+ Monos were generally downregulated, and the representative GO terms are presented in Figure 3E down. The differentially expressed TFs in IFITM2+ Monos (such as STAT1, IRF7, FOS and CEBPD) among the healthy control, pre-treatment, and post-treatment groups are displayed in Figure 3F.

Monocle trajectory analysis was performed to reconstruct and characterize the relationships between the peripheral blood Monos in the different study groups and to derive reprogramming trajectories using an unbiased method (Trapnell et al., 2014; Qiu et al., 2017; Packer et al., 2019). According to the change in trajectory, the Monos progressed toward the post-treatment state from the healthy state during stage 1 induction before bifurcation (pre-branch); the Monos then transformed toward two distinct cell fates: cell fate 1 (pre-treatment state) and cell fate 2 (post-treatment state) (Figure 3G).

We then examined the pseudo-time dynamics of the gene expression patterns in Monos and arranged them into five clusters (Figures 3H, I). The genes in cluster Module 1 (e.g., *PGK1*, *DNAJC15*, *COA6*, *ATP5MG*, *COX6B1*, *COX4I1*) were primarily associated with cellular respiration (oxidative phosphorylation, ATP metabolic process, electron transport chain, ATP synthesis coupled electron transport, aerobic respiration); the expression patterns remained constant under the cell fate 1 condition, however, initially increased and then decreased along the pseudo-time under the cell fate 2 condition (Figures 3H–J). Cluster Module 4 comprised genes related to type I IFN/virus responses, such as *ISG15*, *IFI6*, *IFI44L*, *RSAD2*, *IFITM3*, *IFIT2*, *etc.* The expression of these genes increased from the beginning of reprogramming in the cell fate 1 stage, however, decreased slightly beginning mid-cell fate 2 stage (Figures 3H–J). Cluster Module 5 genes largely comprised genes that were enriched in immune response and phagocytosis, such as *FCGR3B*, *MNDA*, *KPNB1*, *ARPC2*, *ABCA1*, *KIAA1109*, *etc.* These genes were highly expressed under cell fate 1 condition, however, decreased from the beginning of reprogramming under cell fate 2 condition (Figures 3H–J).

The dysregulation of IFNs production and function could mediate immune pathogenesis such as inflammatory autoimmune diseases and infectious diseases (Chen et al., 2017). Furthermore, we leveraged the transcription factor network to unravel that IFITM2+ Monos and CYP4F3+ Monos could acquire inflammatory states *via* the coordinated activity of inflammatory pathways driven by response to type I IFN, cellular response to type I IFN, response to type IFN-γ and type I IFN-mediated signaling pathway (Supplementary Figure S1). IRF7, STAT1, STAT2 and SP100 were key transcription factors associated with the inflammatory or regulatory roles expressed in Monos. Breifly, the type I IFN and IFN-γ signatures were significantly enhanced, accompanied by the transformation of healthy Monos into inflammatory Monos under the pre-teatment state, while the IFN responses recovered to control level in the post-treatment state, suggesting the effective reduction of inflammation in patients with JDM after treatment. Furthermore, cellular respiration is enhanced from the beginning of reprogramming under the cell fate 2 condition.

### 3.3 Re-clustering the peripheral blood T-cell subsets and their pseudo-time trajectory reconstruction

**FIGURE 4:**
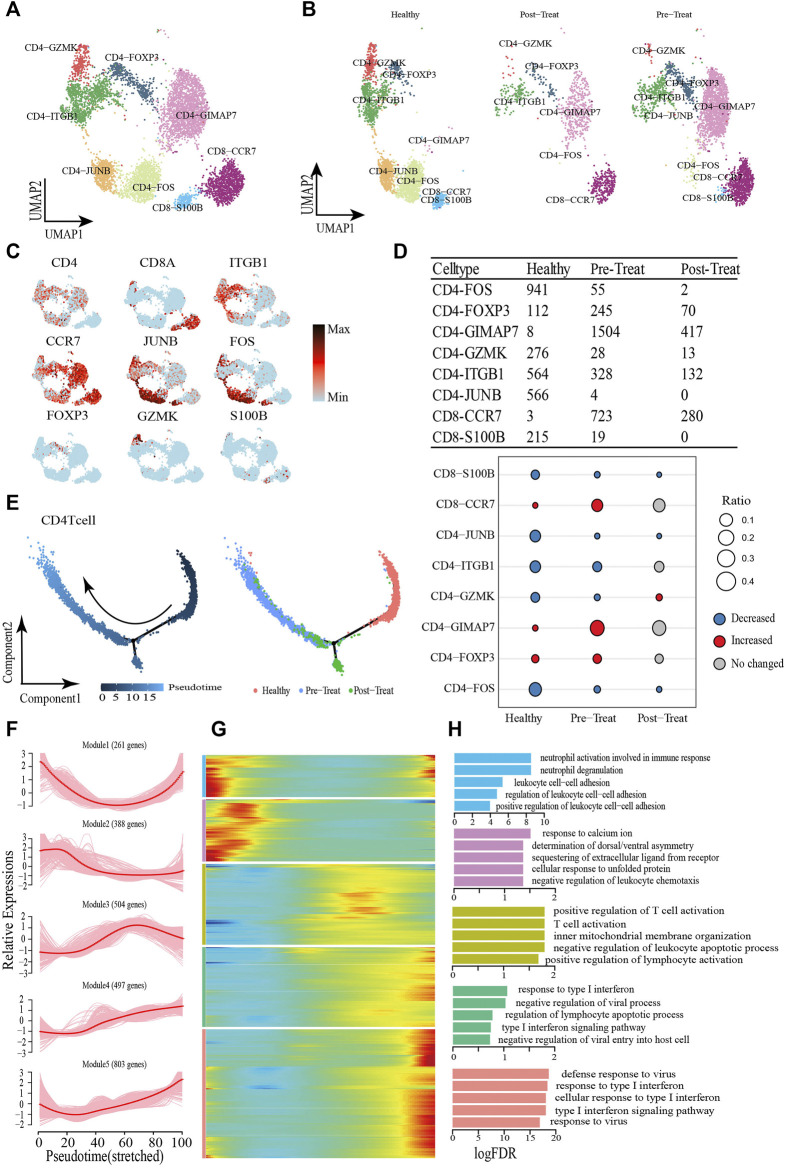
Dynamic change process of T cell in response to JDM disease occurrence and treatment. **(A,B)** Visualization of T cell sub-clusters in human peripheral blood sample *via* Uniform Manifold Approximation and Projection (UMAP) plots by cell identity **(A)** and different stages **(B)**. **(C)** The feature plots of marker genes of T cell sub-clusters. **(D)** Heterogeneity of cell type composition of T cell sub-clustersin healthy control, pre-treatment, and post-treatment groups. The table shows the number of each cell type in each group (up); and the bubble chart represents the changes in the cell ratio of cell types between the pre-treatment and healthy control groups, and between the post- and pre-treatment groups in peripheral blood (down). **(E)** Pseudotime trajectory (Monocle analysis) of the CD4^+^ T cell sub-clusters. Cells are colored based according to the predicted pseudotime (left) and groups (right). **(F)** The expression dynamics of top DEGs were cataloged into five major clusters in a pseudotime manner. **(G)** A heatmap shows the different expression patterns of representative DEGs (cataloged in five clusters) along the reprogramming trajectory. Color key from blue to red indicates relative expression levels from low to high. **(H)** GO enrichment analyses of each gene cluster.

T cells are important for immune response and have been reported as a therapeutic target in MDA5+ DM (Nombel et al., 2021; Wu et al., 2021), and we analyzed the T-cells (including CD4^+^ and CD8^+^ T-cells, excluding CD3^+^ T-cells due to low cell numbers (1 cell) in the healthy control) compartments in the peripheral blood and assessed their heterogeneity with eight subsets. The CD4^+^ T-cells comprised CD4-*ITGB1*, CD4-*JUNB*, CD4-*FOS*, CD4-*FOXP3*, CD4-*GZMK*, and CD4-*GIMAP7* subsets, whereas CD8^+^ T-cells comprised CD8-*S100B* and CD8-*CCR7* subsets (Figures 4A, B, Supplementary Table S4). Representative signature genes are presented in feature plots (Figure 4C). Among the three study groups, the proportions of CD8-*S100B*, CD4-*JUNB*, CD4-*ITGB1*, CD4-*GZMK* and CD4-*FOS* were significantly decreased in the pre-treatment group compared to the healthy controls, while CD8-*S100B* and CD4-*JUNB* were not detected within the post-treatment group (Figure 4D). Moreover, the proportions of CD4-*GIMAP7*, CD4-*FOXP3* and CD8-*CCR7* were significantly increased in the pre-treatment group relative to that in the healthy control group (Figure 4D).

Pseudo-time trajectory analysis was performed to capture T-cells asynchronously transitioning from one transcriptomic state to the next. We first captured a linear trajectory for CD4^+^ T-cells, which progressed from an initially healthy state to post-treatment state and culminated in a pre-treatment state (Figure 4E). To understand the biological processes driving pseudo-time components, we investigated which genes covary in expression with pseudo-time. Consequently, we identified five gene clusters expressed in CD4^+^ T-cells based on the pseudo-time dynamics of their expression patterns (Figures 4F, G). The expression levels of genes in Module 1 (e.g., *SLC2A3* and *CCL5*) were initially downregulated and subsequently upregulated until pseudo-time termination, meanwhile, Module 2 genes (e.g., *JUN*, *DUSP1*, *FOS*) were expressed early. In Module 3, during the pseudo-time mid-phase, we observed highly expressed genes associated with T-cell/leukocyte activation, such as *CCR7* and *Il6ST*. Finally, the genes within Module 4 (e.g., *IFIT1* and *IRF9*) and Module 5 (e.g., *ISG15* and *RSAD2*) were associated with type I IFN/virus responses and were upregulated beginning in the pseudo-time mid-phase, and highly expressed by the pseudo-time end point (Figure 4H).

**FIGURE 5:**
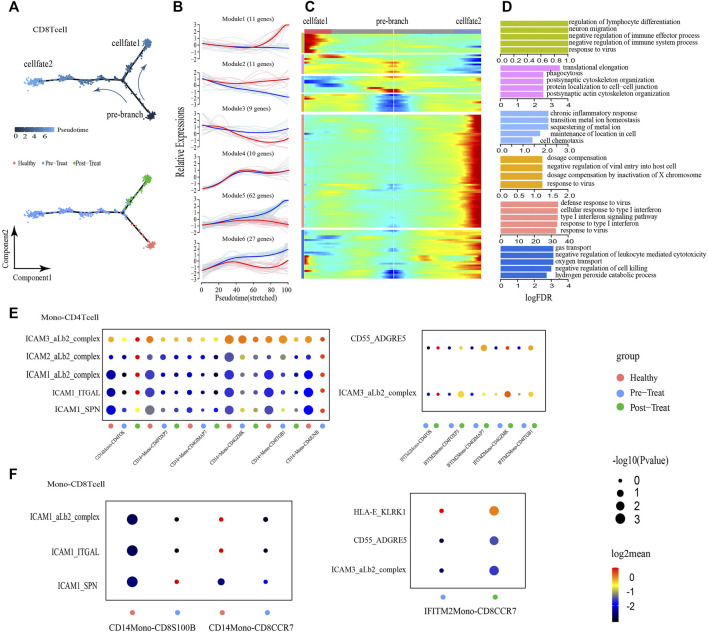
CD8^+^ T cells showed transition from a naive state to an inflammatory state and regulated by non-classical monocytes. **(A)** Pseudotime trajectory (Monocle analysis) of the CD8^+^ T cell sub-clusters. Cells are colored based according to the predicted pseudotime (up) and groups (bottom). **(B)** The expression dynamics of top DEGs were cataloged into six major clusters in a pseudotime manner shown as red lines (cell fate 1) and blue lines (cell fate 2). **(C)** A heatmap shows the different expression patterns of top DEGs (cataloged in six clusters) along the development of Cell fate1 and cell fate 2. **(D)** GO enrichment analyses of each gene cluster. **(E,F)** Selected ligand-receptor interactions (*y*-axis) against cell types (*x*-axis) from monocytes, CD4^+^ T cells **(E)** and CD8^+^ T cells **(F)** of human JMD samples. *p* values are indicated by circle size. The means of the average expression level of interacting molecule 1 in cluster 1 and interacting molecule 2 in cluster 2 are indicated by color.

CD8^+^ T-cells were initially healthy before bifurcation (pre-branch), whereas following bifurcation two distinct branches arose (cell fate 1 and cell fate 2), representing two major cell states (post-treatment and pre-treatment) in the late reprogramming stage (Figure 5A). Six major categories of transcriptional gene clusters were observed in the characterized expression patterns (Figures 5B, C). We then focused on the trajectory of cell fate 2 and found that genes in Modules 1 and 2 were gradually downregulated from the beginning of reprogramming, whereas genes in Module 3 (e.g., *TMSB4X*, *CCL5*, *S100A9*) remained relatively stable during cell fate 2; these genes were largely associated with regulation of biological processes, such as “cell chemotaxis.” In contrast, Module 4 genes (e.g., *IFITM1*, *XIST*, *SEPTIN6*) were gradually upregulated from the beginning of reprogramming and maintained at high expression levels until the final stage. Finally, Module 5 and Module 6 genes were upregulated along the pseudo-time, among which Module 5 genes (e.g., *IFI44L*, *ISG15*, *IFIT3*) were predominantly associated with type I IFN/virus responses and Module 6 genes (e.g., *HBB*, *IL7R*, *HBA2*) were largely involved in the regulation of biological processes, such as “oxygen transport” (Figure 5D). When focusing on cell fate 1, we found that the expression of genes in Module 5 and Module 1 exhibited the greatest differences compared with cell fate 2. That is Module 5 genes exhibited slight upregulation from the beginning and then subsequently became downregulated. Meanwhile, genes in Module 1 (e.g., *ZBTB16*, *NDFIP1*, *PCBP2*) were upregulated at the end of reprogramming with predominant involvement in the GO term “negative regulation of immune system process.” According to these results, the type I IFN signature was also significantly enhanced, accompanied by the transformation of healthy T-cells into inflammatory T-cells (CD4^+^ and CD8^+^ T-cells), whereas their normal functions like “cell-cell adhesion” were downregulated under the inflammatory state.

### 3.4 Cellular interactions between Monos and T-cells in the peripheral blood

To explore the roles of Monos in the progression of JDM, we employed an unbiased ligand-receptor interaction analysis of the Monos and T-cell subsets *via* communication calculations with CellphoneDB, with the aim of characterizing the interactions in unambiguously functional clusters (Figures 5E, F). We captured two pairs of ligand-receptor interactions between IFITM2+ Monos and CD4^+^ T-cell subsets that differed significantly between the pre- and post-treatment groups. ICAMs are intercellular adhesion molecules that mediate inflammation (Lyck and Enzmann, 2015; Shen et al., 2018). We also identified five pairs of ligand-receptor interactions that associated with inflammation between CD14^+^ Monos (normal Monos) and CD4^+^ T-cell subsets (ICAM3-aLb2 complex, ICAM2-aLb2 complex, ICAM1-aLb2 complex, ICAM1-ITGAL, ICAM1-SPN) that occurred primarily in the healthy state and were lost in the pre- and post-treatment states (Figure 5E).

Moreover, the CD55-ADGRE5 and ICAM3-aLb2 complex interactions between IFITM2+ Monos (inflammatory Monos) and CD4^+^ T-cell subsets enhanced their interactions in the post-treatment state compared to the pre-treatment state. In line with the interactions between Monos and CD4^+^ T-cell subsets, similar interaction patterns were observed between Monos and CD8^+^ T-cell subsets (Figure 5F). Collectively, these findings illustrate the molecular basis for the potential cellular interactions between Monos and T-cells, and found that inflammatory-related monocytes could regulate T-cells in the peripheral blood of patients with JDM.

### 3.5 Distinct functional profiles of the SMC subsets in muscle cells and their pseudo-time trajectory reconstruction

**FIGURE 6:**
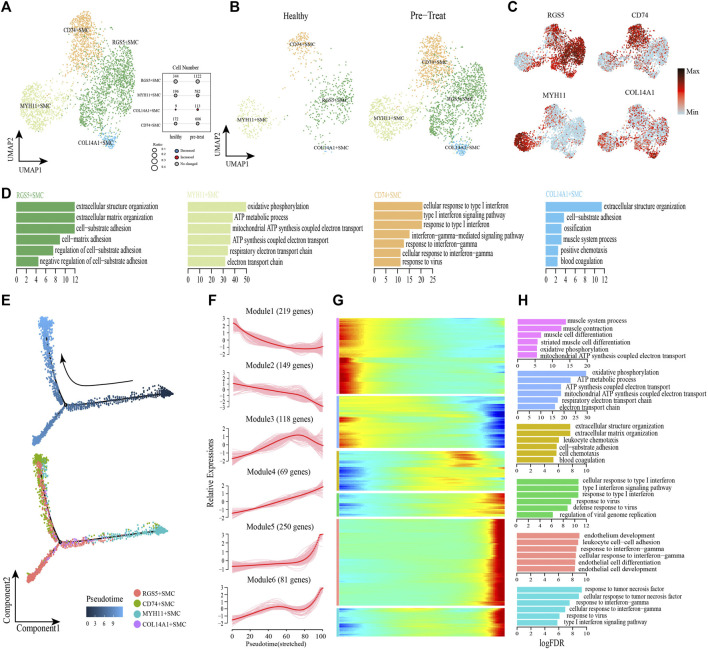
Gene expression profile analysis of smooth muscle cells in JDM patients. **(A,B)** Visualization of smooth muscle cell sub-clusters in human muscle sample *via* Uniform Manifold Approximation and Projection (UMAP) plots by cell identity **(A)** and different stages **(B)**. **(C)** The feature plots of marker genes of smooth muscle cell sub-clusters. **(D)** GO enrichment analyses of marker genes of smooth muscle cell sub-clusters. **(E)** Pseudotime trajectory (Monocle analysis) of the smooth muscle cells. Cells are colored based according to the predicted pseudotime (top) and groups (bottom). **(F)** The expression dynamics of top DEGs were cataloged into six major clusters in a pseudotime manner. **(G)** Gene expression heatmap of top DEGs (cataloged in six clusters) in a pseudo-temporal order. **(H)** GO enrichment analyses of each gene cluster.

To describe the functional profile of SMCs, one type of the muscle stromal cells, four subsets were identified by re-clustering according to their differentially expressed markers (Figures 6A–C, Supplementary Table S5). The GO annotation of marker genes in CD74^+^ SMCs (e.g., *HLA-E*, *HLA-DRB1*, *CD74*) showed their capacity to respond to IFN and viruses (e.g., cellular response to type I IFN, cellular response to IFN-γ, response to viruses, *etc.*) (Figure 6D). The marker genes expressed in COL14A1+ SMCs (e.g., *CD44*, *IGFBP5*, *ADRA2A*) were largely involved in the regulation of biological processes, such as “extracellular structure organization,”“cell-substrate adhesion,” “muscle system process,” “ossification,” “positive chemotaxis,” and “blood coagulation” (Figure 6D). MYH11+ SMCs preferentially expressed marker genes (e.g., *NDUFA4*, *COX6C*, *COX7C*) involved in ATP synthesis and cellular respiration (Figure 6D). Meanwhile, genes expressed in RGS5+ SMCs (e.g., *CD36*, *COL3A1*, *SPARC*) were predominantly responsible for cell-substrate adhesion (Figure 6D).

Next, monocle trajectory analysis was performed to reconstruct and characterize the relationships among the SMC lineages and to derive pseudo-time trajectories. The monocle pseudo-time analysis results inferred that the direction of the transition from healthy to pre-treatment transcriptomic state began with the MYH11+ SMCs and ended with the CD74^+^ SMCs as the terminal cluster (Figure 6E). To gain insights into gene expression dynamics along this trajectory, we assessed six major categories of transcriptional gene clusters based on characterized expression patterns (Figures 6F, G). The expression of genes in Module 1 (e.g., *PLN*, *MYH11*, *SORBS2*) and Module 2 (e.g., *COX7C*, *COX5B*, *ATP5ME*) were downregulated from the beginning of the reprogramming trajectory, among which Module 1 genes were largely related to the muscle system process, and Module 2 genes were mainly associated with ATP metabolism and cellular respiration (Figure 6H). We observed that genes in Module 3 (e.g., *COL3A1*, *CD36*, *TIMP1*) were highly expressed during the mid-late pseudo-time period, and were largely involved in the regulation of biological processes, such as “extracellular structure organization,” “cell-substrate adhesion,” “leukocyte chemotaxis,” “cell chemotaxis,” and “blood coagulation” (Figure 6H). Module 4 genes (e.g., *IFI27*, *HLA-A*, *IFIT1*) were upregulated from the beginning along the reprogramming trajectory, and genes in Module 5 (e.g., *CD74*, *HLA-DRB1*, *TRIM22*) and 6 (e.g., *CCL2*, *HLA-E*, *IFIT3*) were upregulated by the pseudo-time end point; genes in these three clusters were predominantly responsible for the regulation of immune defense and responses to IFNs/virus (Figure 6H).

In addition, transcription factor network shows that the CD74^+^ SMCs, which highly expressed inflammatory transcription factors SP100, EGR1 and IRF7, could achieve inflammatory states by coordinating activity on inflammatory pathways driven by response to IFN-γ and type I IFN-mediated signaling pathway (Supplementary Figure S2). These results revealed that CD74^+^ SMCs were associated with inflammatory state in muscle, and both type I IFN and IFN-γ signatures were significantly enhanced when healthy SMCs were transformed into inflammatory SMCs, accompanied by dysfunction of processes in the muscle system and cellular respiration.

### 3.6 Distinct functional profiles of the fibroblast subsets in muscle cells and their pseudo-time trajectory reconstruction

**FIGURE 7:**
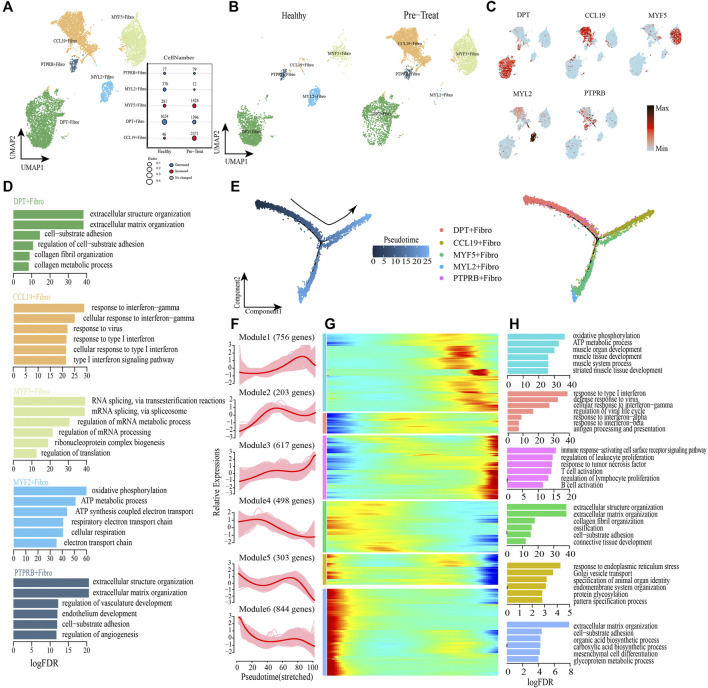
Fibroblasts with inflammatory gene expression profiles emerge in muscle samples of JDM patients. **(A,B)** Visualization of fibroblast sub-clusters in human muscle sample *via* Uniform Manifold Approximation and Projection (UMAP) plots by cell identity **(A)** and different stages **(B)**. **(C)** The feature plots of marker genes of fibroblast sub-clusters. **(D)** GO enrichment analyses of marker genes of fibroblast sub-clusters. **(E)** Pseudotime trajectory (Monocle analysis) of the fibroblast. Cells are colored based according to the predicted pseudotime (left) and groups (right). **(F)** The expression dynamics of top DEGs were cataloged into six major clusters in a pseudotime manner. **(G)** Gene expression heatmap of top DEGs (cataloged in six clusters) in a pseudo-temporal order. **(H)** GO enrichment analyses of each gene cluster.

Next, we performed unsupervised re-clustering of fibroblasts (another type of muscle stromal cells) and observed further heterogeneity with five subsets, each containing unique marker gene profiles (Figures 7A–C, Supplementary Table S6). Among these subsets, CCL19+ fibroblasts preferentially expressed genes (e.g., *CCL19*, *HLA-DRB1*, *ISG20*, *IFITM2*) involved in IFN and virus responses (e.g., cellular response to IFN-γ, type I IFN signaling pathway, response to virus, *etc.*) (Figure 7D); a significant increase was observed in the proportion of this cell subtype in the pre-treatment group compared with the control group (Figure 7A). The marker genes expressed in DPT + fibroblasts (e,g., *DPT*, *APOD*, *COL1A2*) were primarily associated with regulation of biological processes, such as “extracellular matrix organization,”“cell-substrate adhesion,” “collagen fibril organization,” and “collagen metabolic process” (Figure 7D). MYF5+ fibroblasts highly expressed genes associated with regulation of RNA splicing and mRNA metabolic processes, such as *MYOD1*, *HNRNPA1*, *VIM*, *NPM1*, *etc.* (Figure 7D), while MYL2+ fibroblasts highly expressed genes associated with the regulation of ATP synthesis and cellular respiration, such as *COX6A2*, *COX7A1*, *ATP5F1D*, *MYL2*, *etc.* (Figure 7D). Additionally, genes highly expressed in PTPRB + fibroblasts (e.g., *VWF*, *AQP1*, *CDH5*) were enriched in “extracellular matrix organization,”“cell-substrate adhesion,” “endothelium development,” and “regulation of angiogenesis” (Figure 7D).

Monocle trajectory analysis was then performed to reconstruct and characterize the relationships among fibroblast subsets and to derive pseudo-time trajectories. The transcriptomic states of fibroblasts began with DPT + fibroblasts and PTPRB + fibroblasts and were partitioned into two distinct states: MYF5+ fibroblasts and MYL2+ fibroblasts were terminal clusters of one end state, while the CCL19+ fibroblasts represented the terminal cluster of the other end-state (Figure 7E). To gain insights into the gene expression dynamics occuring from healthy to inflammatory states, we observed six major transcriptional gene clusters based on their characteristic expression patterns (Figures 7F, G). Genes in Module 4 (e,g., *DPT*, *COL3A1*, *FN1*), Module 5 (e,g., *JUN*, *GAS1*, *FGFR1*) and Module 6 (e,g., *HSPG2*, *ALDH1A2*, *SPARCL1*) were expressed early and subsequently downregulated along the reprogramming trajectory, among which Module 4 genes were largely associated with regulation of collagen metabolism and cell-substrate adhesion, whereas Module 5 and Module 6 genes were predominantly responsible for regulation of “response to endoplasmic reticulum stress” and “extracellular matrix organization” respectively (Figure 7H). Subsequently, genes enriched in Module 2 (e.g., *ISG15*, *IFITM1*, *HLA-A*) were upregulated during mid-stage and retained high expression until the end of the pseudo-time; these genes were involved in antigen presentation and responses to IFNs/virus (Figure 7H). Module 1 genes (e.g., *UQCRB*, *TNNI1*, *ACTA1*, *etc.*) were highly expressed during the mid-to-late stage of the reprogramming pseudo-time and were primarily associated with the regulation of muscle development and cellular respiration (Figure 7H). Finally, we observed that genes in Module 3 (e.g., *CD74*, *HLA-DRA*, *HLA-DRB1*) were upregulated at the end of the reprogramming pseudo-time and were responsible for activation of the immune system, including “regulation of biological processes of the immune response-activating cell surface receptor signaling pathway,” “regulation of leukocyte proliferation,” “response to tumor necrosis factor,” “T-cell activation,” “regulation of lymphocyte proliferation,” and “B cell activation” (Figure 7H).

As before, transcription factor network analysis was performed and result has shown that inflammatory transcription factors EGR1 and IRF7 were highly expressed in CCL19+ fibroblasts and drove them to inflammatory states by pathways of response to IFN-γ and type I IFN-mediated signaling pathway (Supplementary Figure S3). In summary, CCL19+ fibroblast was one of the inflammatory cell subtype in muscle, and the IFN signatures (type I IFN and IFN-γ) exhibited a significant enhancing trend in muscle fibroblasts when transforming from a healthy state to an inflammatory state, accompanied by the activation of immune defense and dysfunction of collagen metabolism. Moreover, the genes associated with muscle development and cellular respiration (Module 1) were upregulated during the mid-to-late stage of the fibroblasts’ transition to an inflammatory state, which differed from the downregulated expression of related genes in SMCs.

### 3.7 Cellular interactions of immune cells and functional cells in muscles between the healthy control and pre-treatment group

**FIGURE 8:**
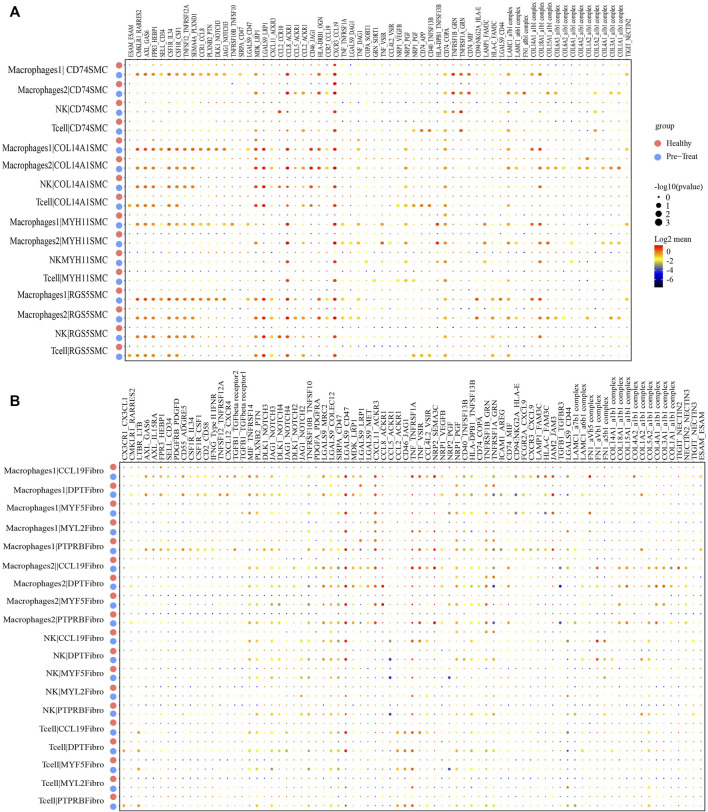
Cellular interaction analysis between immune cells and smooth muscle cells or fibroblasts in JDM muscle samples. **(A, B)** Bubble chart showing ligand-receptor relationship between immune cells and smooth muscle cells **(A)** or fibroblasts **(B)**.

As so far, the infiltration of specific immune cell subsets was one of the characterizations of inflammatory process in JDM (Wienke et al., 2018). Therefore, we performed an unbiased ligand-receptor interaction analysis to explore the relationship between immune cells and SMCs or fibroblasts in the muscle (Figures 8A, B). We captured 39 pairs of ligand-receptor interactions between immune cells and CD74^+^ SMCs that significantly differed between the healthy control and pre-treatment groups. Among these, 24 pairs were found between macrophages 1 and CD74^+^ SMCs (such as CCL5-ACKR1), 18 pairs were found between macrophages 2 and CD74^+^ SMCs, 8 pairs between NK cells and CD74^+^ SMCs, and 10 pairs between T-cells and CD74^+^ SMCs, that were enhanced in the pre-treatment state compared to the healthy state (Figure 8A). Moreover, 44 pairs of ligand-receptor interactions were detected between immune cells and COL14A1+ SMCs, 33 pairs between immune cells and MYH11+ SMCs, and 45 pairs between immune cells and RGS5+ SMCs that exhibited significant enhancement within the inflammatory state.

Similar results were observed for immune cells and fibroblasts. Eighteen pairs of ligand-receptor interactions between immune cells and CCL19+ fibroblasts (Figure 8B), 14 pairs between immune cells and DPT + fibroblasts, 10 pairs between immune cells and MYF5+ fibroblasts, 12 pairs between immune cells and MYF2+ fibroblasts, and 13 pairs between immune cells and PTPRB + fibroblasts were captured and primarily occurred during the inflammatory state. These results suggest that immune cells, including macrophages, NK cells and T-cells infiltrate muscle tissue during the inflammatory state.

## 4 Discussion

Current data regarding the changes within the immune system associated with JDM pathogenesis and the response to treatment is lacking. In this study, changes in cell composition, dynamics in gene expression patterns at the cellular level, and immunological features of patients with JDM pre- and post-treatment were described using scRNA-seq. We identified a total of nine and seven distinct cell types in the peripheral blood and muscle, respectively, and demonstrated the heterogeneity of patient-specific cell populations in JDM. Furthermore, overactivation of the type I IFN response pathway was associated with untreated JDM patients in all cell types. Finally, the non-classical-Monos were found to regulate T-cells in peripheral blood while immune cell infiltration was detected within muscle tissue under inflammatory conditions. These results provide novel insights into the cellular mechanisms underlying JDM pathogenesis as well as potential targets for therapeutic intervention.

The activation of type I IFN signaling in JDM blood and target tissues has been reported in many previous studies (Wenzel et al., 2006; Baechler et al., 2007; Bilgic et al., 2009; Baechler et al., 2011; Wong et al., 2012; Neely et al., 2019; Soponkanaporn et al., 2019; Neely et al., 2022), and has been identified as a predominant feature in MDA5^+^ DM patients that is related to endothelial injury (Funauchi et al., 2006; He et al., 2021), vasculopathy (Ono et al., 2019; Cassius et al., 2020), and lung injury (Nakano et al., 2012; Takada et al., 2015; Ye et al., 2022). Type I IFN plays essential role in establishing and modulating host immune response to the complex pathogenic or environmental stimuli *via* induction of IFN-stimulated genes (ISGs) through Janus kinase (JAK)–signal transducer and activator of transcription (STAT) signaling pathway, and the dysregulation of type I IFN production and function could induce an inflammatory state in patients by aberrantly activating inflammatory responses or improperly suppressing microbial controls (Chen et al., 2017). Our study confirmed a significantly enhanced type I IFN response in peripheral blood Monos and T-cells, as well as muscle SMCs and fibroblasts from untreated patients with JDM. For example, the type I IFN-related TFs *IRF1*/*2*/*7* and *STAT1* were highly enriched in the CD14^+^ Monos, while *IRF1*/*2*/*7* and *STAT1*/*2* were enriched in the IFITM2+ Monos of these patients (Figure 3F). Furthermore, the DEGs associated with type I IFN response, such as *ISG15*, *IFI6*, *IFITM3* and *IFIT2* in peripheral blood Monos; *IFIT1*, *IRF9, ISG15* and *RSAD2* in peripheral CD4^+^ T-cells; *IFI44L*, *ISG15* and *IFIT3* in peripheral CD8^+^ T-cells; *IFI27*, *HLA-A*, *IFIT1* in muscle SMCs; and *ISG15*, *IFI6*, *HLA-A*/*B*/*C* in muscle fibroblasts, were overexpressed in the inflammatory state. Among these genes, *ISG15* is one of the most strongly induced ISGs upon exposure to type I IFN, virus, lipopolysaccharide (LPS), and other stresses (Liu et al., 2004; Jeon et al., 2010). Considering that type I IFNs play critical roles in innate immune responses regulating the antiviral responses, there is no doubt that *ISG15* and its conjugation to target proteins play critical roles in the type I IFN-induced immune responses. Notably, type I IFN has been reported to promote CD8^+^ T-cell expansion (Kolumam et al., 2005), while ISG15+ CD8^+^ T-cells may represent a promising prognostic biomarker in MDA5^+^ DM (Ye et al., 2022). In this study, we also observed the expansion of CD8^+^ T-cells in untreated patients with JDM, further confirming that ISG15+ CD8^+^ T-cells might represent prognostic biomarker for JDM.

The data of the peripheral blood monocyte compartment in this study exhibited high dysregulation in JDM patients. Three subsets of peripheral blood monocytes, among which only CD14^+^ monocytes were found in healthy control, while the other two monocytes (IFITM2+ Monos and CYP4F3+ Monos) were largely produced in patients with JDM. Previous work suggests that CD16^+^ monocytes are non-classical-monocytes, and this cell type has traditionally been thought to be immune-regulatory and plays an important role in autoimmune diseases (Narasimhan et al., 2019). In fact, Neely et al. identified a CD16^+^ monocyte subcluster that was skewed toward an inflammatory and antigen-presenting phenotype in JDM (Neely et al., 2022). Meanwhile, Mukherjee et al. found a similar inflammatory CD16^+^ non-classical-monocyte population in adult systemic lupus erythematosus (SLE) peripheral blood (Mukherjee et al., 2015). In our study, IFITM2+ Monos and CYP4F3+ Monos, both of which were highly expressed *CD16*, represented inflammatory populations as significantly enhanced inflammatory associated type I IFN response pathways were observed within the pre-treatment state. In contrast, the type I IFN response was downregulated accompanied by the disappearance of CYP4F3+ Monos in the post-treatment state compared to pre-treatment, suggesting that CYP4F3+ Monos was more relevant to inflammation than IFITM2+ Monos. Meanwhile, the skin rash was basically subsided, and the muscle strength recovered normally of the patients after treatment, revealing the effective reduction of inflammation by treatment (treated with prednisone, hydroxychloroquine, and cyclophosphamide for 3–6 months). Therefore, CYP4F3+CD16^+^ monocytes may also represent a potential prognostic biomarker for JDM.

Cell type composition heterogeneity and transcriptional profile changes were also observed in muscle cells of untreated patients with JDM and healthy controls. CD74^+^ SMCs and CCL19+ fibroblasts were identified as inflammatory-related cell subtypes in patients with JDM as these cells expressed various gene markers involved in the type I IFN signaling pathways, such as *HLA-E*, *EGR1*, *ISG20* and *IFITM2*. Moreover, these 2 cell subtypes were terminal clusters of SMCs and fibroblasts, respectively, on the trajectory of their transition from a healthy to an inflammatory state. The transcription factor networks confirmed that both CD74^+^ SMCs and CCL19+ fibroblasts could achieve inflammatory states by coordinating activity on inflammatory pathways driven by response to IFN-γ (related DEGs such as *HLA-A*/*B*/*C*, *IRF7*) and type I IFN-mediated signaling pathway (Supplementary Figures S2, S3). Prior reports show that the increasing IFN-γ signature was also observed in polymyositis and dermatomyositis complicated by rapidly progressive or chronic interstitial lung disease (Gono et al., 2014; Ishikawa et al., 2018). Indeed, high levels of IFN-γ have been implicated in the development and severity of MDA5+ DM (Ye et al., 2022), and been shown to induce proinflammatory CX3CL1 in lung fibroblasts (Isozaki et al., 2011).

Meanwhile, transcription factor networks also showed that EGR1 (early growth response factor 1) and IRF7 (interferon regulatory factor 7) play central roles in the inflammation in both CD74^+^ SMCs and CCL19+ fibroblasts, therefore, inhibition of the activities of EGR1 and/or IRF7 may contribute to reducing inflammation in muscle. EGR1 has been identified as potential therapeutic target to inhibit the inflammation induced by cholestasis for cholestatic liver injury (Zhang et al., 2019). Ho et al. (2016) highlighted the integrative role of EGR1 in renal inflammation and fibrosis, and suggested that EGR1 may be a therapeutic target for human kidney diseases (Ho et al., 2016). While IRF7 has emerged as the crucial regulator of type I IFNs against pathogenic infections, therefore, the tight regulation of IRF7 expression and activity is imperative in dictating appropriate type I IFN production for normal IFN-mediated physiological functions (Ning et al., 2011). Hence, EGR1 and IRF7 may serve as potential therapeutic targets for JDM. In addition, the proportion of CCL19+ fibroblasts increased significantly in JDM patients relative to healthy controls (Figure 7A). Although no significant changes in the proportion of CD74^+^ SMCs were found in this study, we suggest that the proportion changes of CD74^+^ SMCs and CCL19+ fibroblasts in patients may reflect the progression of JDM.

The immune activation signature was also found in muscle cells of untreated patients with JDM, and immune defense-related DEGs (e.g., *CCL2*/*CXCL10*/*HLA-E*/*CCL8*/*HLA-DRB1* in muscle SMCs and *CD74*/*HLA-DRB1*/*HLA-DRA*/*CCL19* in muscle fibroblasts) were activated at the end of the reprogramming trajectory. Among these genes, serum *CXCL10* serves as a biomarker for disease activity in patients with JDM (Wienke et al., 2019). *CXCL10* binds to its receptor CXC chemokine receptor 3 (*CXCR3*) and promotes an inflammatory microenvironment by activating and recruiting various immune cells (Kim et al., 2014). *CXCL10* is also reportedly involved in pathological muscle conditions, such as inflammatory myopathies, suggesting that it serves as a potential therapeutic target for these conditions (Crescioli et al., 2012). Furthermore, all pairs of ligand-receptor interactions between immune cell (including macrophages1, macrophages2, NK, and T-cells) and SMC or fibroblastshowed a significant enhancement in interactions in the inflammatory state (Figures 8A, B), suggesting that immune cells infiltrate muscle tissue in patients with JDM.

Mitochondrial dysfunction has been observed in muscle biopsies of adult patients with dermatomyositis, and a link has been described between type I IFN and mitochondrial dysfunction (Meyer et al., 2017). We also observed mitochondrial dysfunction in muscle SMCs, as enrichment of terms associated with oxidative phosphorylation, ATP metabolic process, and mitochondrial ATP synthesis-coupled electron transport were downregulated in the inflammatory state, accompanied by downregulation of normal functions associated with the muscular system (Figures 6F–H). Consistent with these transcriptomic changes, clinical representations of uneven sizes of muscle fibers (5–30 μm in diameter), the myocyte myofibrillar fibers were partially disordered, and the local myofibrillar fibers were torn, dissolved, and myomere disappeared were observed in JDM patients without treatment. Furthermore, the observed upregulation in cellular respiration helps reduce inflammation in the post-treatment group, indicating a similar mitochondrial dysfunction in the inflammatory state within peripheral blood Monos (Figures 3H–J). However, genes associated with muscle development and cellular respiration were activated at the mid-to-late stage of fibroblasts’ transition to an inflammatory state (Figures 7G, H), which warrants further investigation.

## 5 Conclusion

The scRNA-seq datasets covering the peripheral blood cells and muscle cells have described immune characteristics in patients with JDM. The representative type I IFN signature was found in all the detail analyzed cell types, and IFN-γ signature was also arose in all the cell types except T cells in inflammatory state. IFITM2+ and CYP4F3+ monocytes in peripheral blood, and CD74^+^ SMCs and CCL19+ fibroblasts in muscle were identified as inflammatory-related cell subtypes in JDM patients, and they could acquire inflammatory states *via* the coordinated activity of inflammatory pathways, especcially driven by response to IFN-γ and type I IFN-mediated signaling pathway. EGR1 and IRF7 were identified as key transcription factors associated with the inflammatory or regulatory roles, and may serve as potential therapeutic targets for JDM. The identified cellular and molecular abnormalities provide novel insights into the immunopathogenesis of JDM. Hence, collectively, our data provide a critical resource and important insights into the pathogenesis of JDM that will aid in the development of effective therapeutics. However, this study also has limitations that are important to recognize, such as the limited numbers of peripheral blood and muscle samples available from JDM patients and healthy control, which decreases our power to assess how heterogeneous the transcriptomes are in untreated patients.

## Data Availability

The raw data presented in the study are deposited at the National Genomics Data Center, the Genome Sequence Archive for human repository (https://ngdc.cncb.ac.cn/gsa-human. The accession number is HRA004355).

## Supplementary material

SUPPLEMENTARY MATERIAL S1 Clinical characteristics of 4 patients.

SUPPLEMENTARY FIGURE S1 The network for the inflammatory transcriptional regulation of IFITM2+ Monos and CYP4F3+ Monos.

SUPPLEMENTARY FIGURE S2 The network for the inflammatory transcriptional regulation of CD14+ SMCs.

SUPPLEMENTARY FIGURE S3 The network for the inflammatory transcriptional regulation of CCL19+ fibroblasts.

SUPPLEMENTARY TABLE S1 Marker genes for distinct cell types in peripheral blood.

SUPPLEMENTARY TABLE S2 Marker genes for distinct cell types in muscle.

SUPPLEMENTARY TABLE S3 Marker genes for distinct cell subsets of Monos in peripheral blood.

SUPPLEMENTARY TABLE S4 Marker genes for distinct cell subsets of T cells in peripheral blood.

SUPPLEMENTARY TABLE S5 Marker genes for distinct cell subsets of SMCs in muscle.

SUPPLEMENTARY TABLE S6 Marker genes for distinct cell subsets of fibroblasts in muscle.
