# Supplementary figures and images for: Single-cell profiling of peripheral blood and muscle cells reveals inflammatory features of juvenile dermatomyositis
Source: Front Cell Dev Biol. 2023 Apr 20;11:1166017. doi: 10.3389/fcell.2023.1166017 (PMC10157079; doi:10.3389/fcell.2023.1166017)

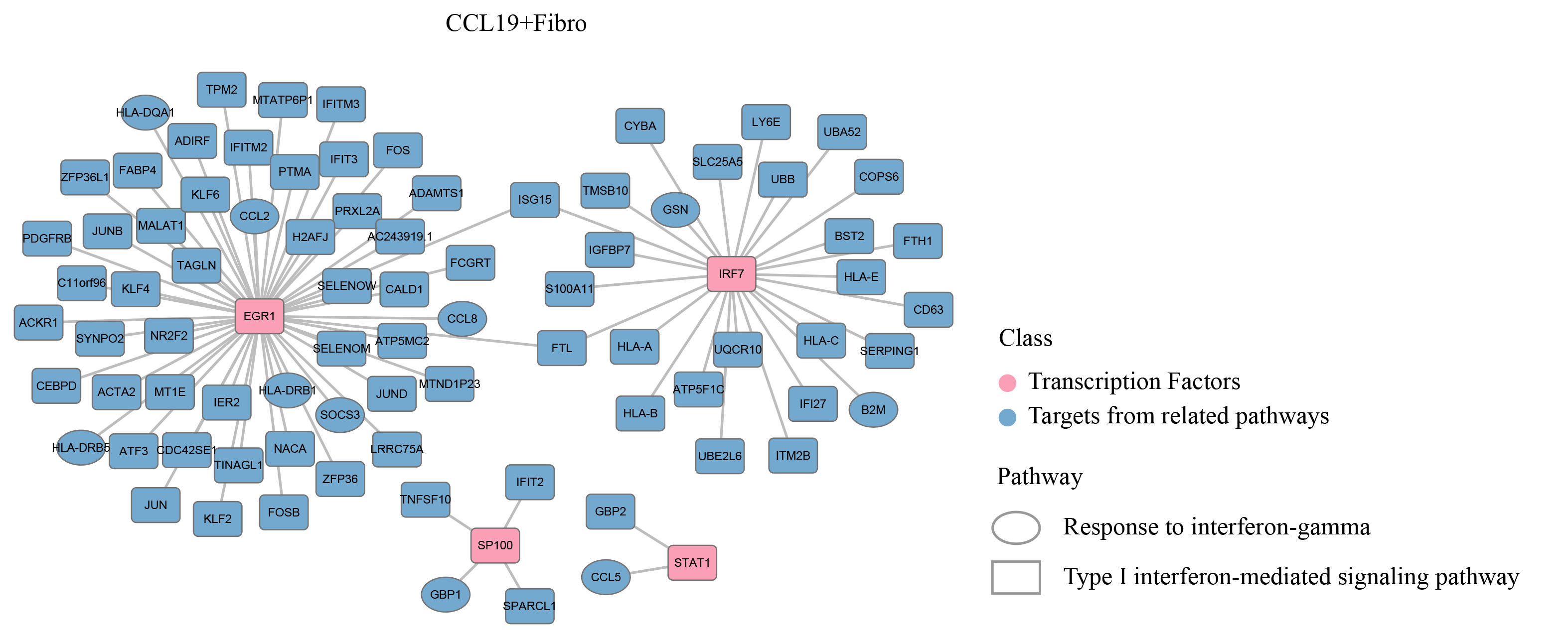

Supplement: Supplementary file 2 [file Image3.JPEG]

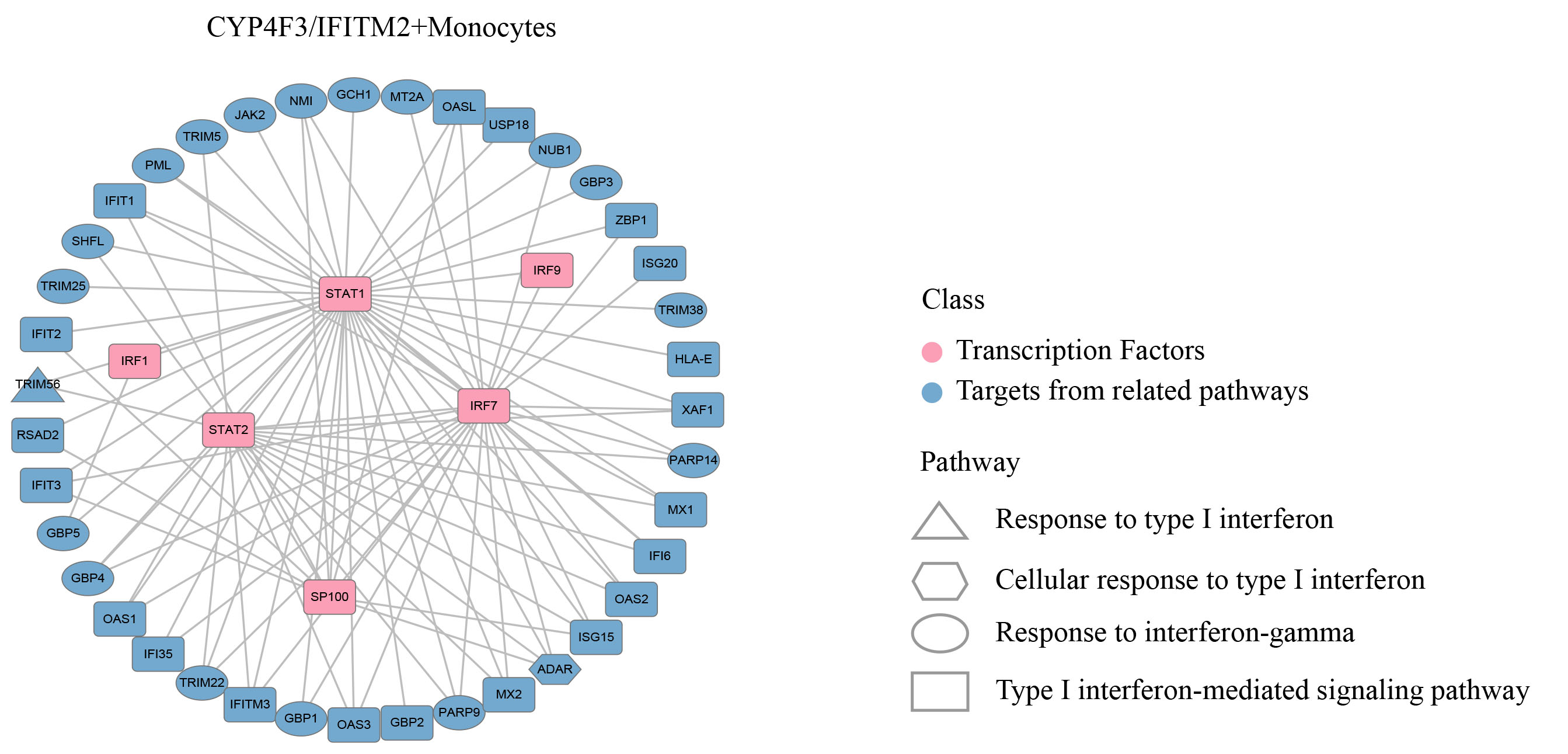

Supplement: Supplementary file 6 [file Image1.JPEG]

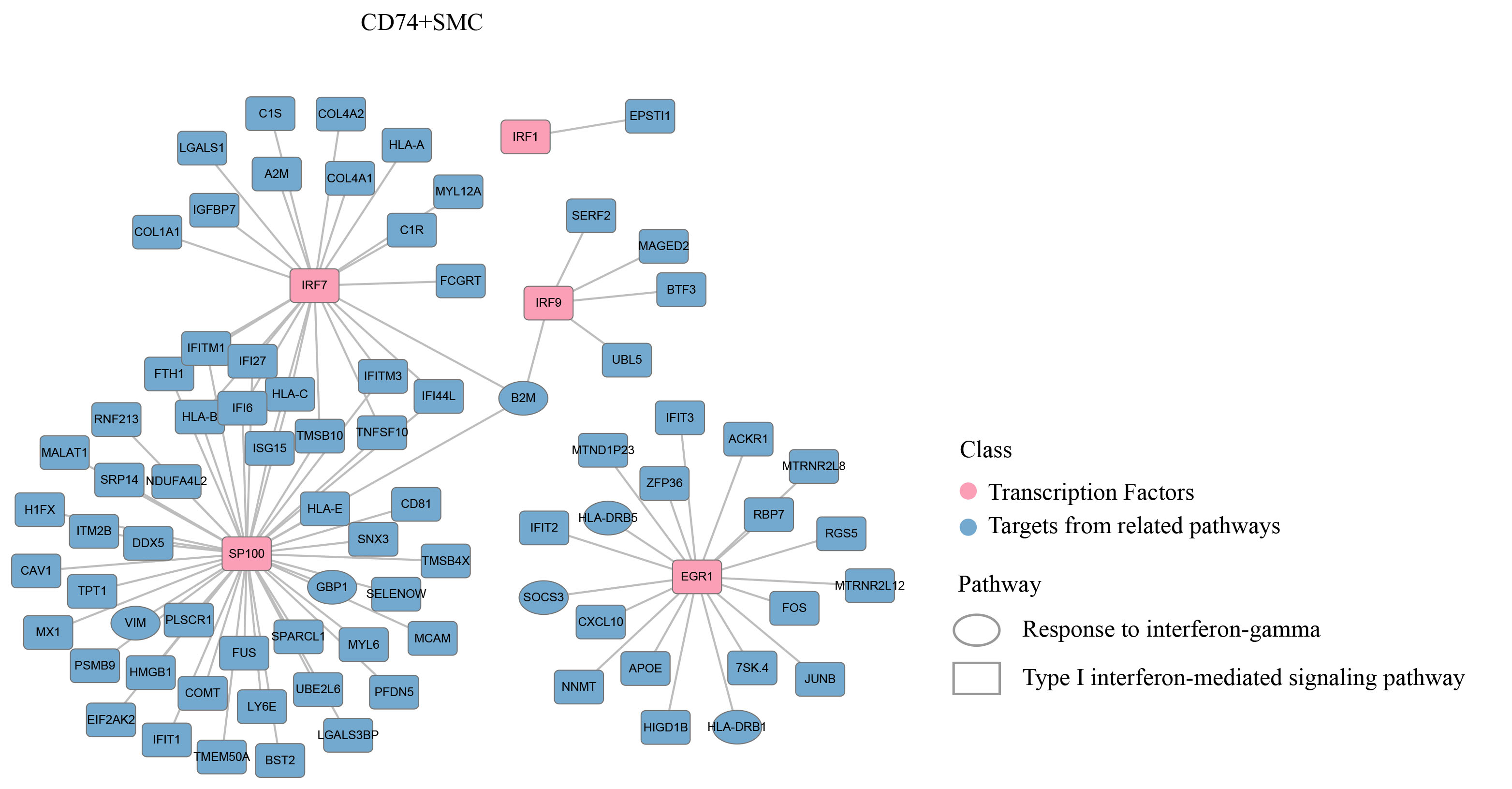

Supplement: Supplementary file 8 [file Image2.JPEG]
