# Supplementary material for: Single-cell profiling of peripheral blood and muscle cells reveals inflammatory features of juvenile dermatomyositis
Source: Front Cell Dev Biol. 2023 Apr 20;11:1166017. doi: 10.3389/fcell.2023.1166017 (PMC10157079; doi:10.3389/fcell.2023.1166017)
Supplement: Supplementary file 10 [file DataSheet1.PDF]

Figure legends

Figure 1. Global transcriptomic profiles of peripheral blood cells from control and JDM patients reveals their cellular subpopulations as determined by ScRNA-seq.

(A) Overall workflow for cell sorting and single-cell data analyses. (B) Uniform Manifold Approximation and Projection (UMAP) plots of single-cell transcriptomic profiles showing cell types of peripheral blood cells from all the samples. Each dot represents a cell, which is colored according to cell type. (C) UMAP plots of single-cell transcriptomic profiles showing cell types of peripheral blood cells from healthy, pre-treatment, and post-treatment groups, respectively. Each dot represents a cell, which is colored according to cell type. (D) Bubble chart of markers for each identified cell type. (E) Feature plots of expression distribution for representative genes for each cell type in peripheral blood. Expression levels for each cell are color-coded and overlaid into UMAP plot. (F) Heterogeneity of cell type composition in healthy control, pre-treatment, and post-treatment groups. The table shows the number of each cell type in each group (up); and the bubble chart represents the changes in the cell ratio of cell types between the pre-treatment and healthy control groups, and between the post- and pre-treatment groups in peripheral blood (down). (G) The number of cells, and box plots of the number of UMIs and genes (with the box plot center, box, whiskers, and points corresponding to the median, interquartile range, 1.5× interquartile range, and outliers, respectively) of Monos (a conbined cell type of monocytes and macrophages) and T cells in each sample of peripheral blood.

Figure 2. Global transcriptomic profiles of muscle cells from control and JDM patients reveals their cellular subpopulations as determined by ScRNA-seq.

(A) Uniform Manifold Approximation and Projection (UMAP) plots of single-cell transcriptomic profiles showing cell types of muscle cells from all the samples. Each dot represents a cell, which is colored according to cell type. (B) UMAP plots of single-cell transcriptomic profiles showing cell types of muscle cells from healthy and pre-treatment groups, respectively. Each dot represents a cell, which is colored according to cell type. (C) Bubble chart of markers for each identified cell type. (D) Feature plots of expression distribution for representative genes for each cell type in muscle. Expression levels for each cell are color-coded and overlaid into UMAP plot. (E) Heterogeneity of cell type composition in healthy control and groups. The table shows the number of each cell type in each group (up); and the bubble chart represents the changes in the cell ratio of cell types between the pre-treatment and healthy control groups in muscle (down). (F) The number of cells, and box plots of the number of UMIs and genes (with the box plot center, box, whiskers, and points corresponding to the median, interquartile range, 1.5× interquartile range, and outliers, respectively) of SMCs and fibroblasts in each sample of muscle.

Figure 3. Classical monocytes were differentiated into non-classical monocytes in circulation of JDM patients.

(A-B) Visualization of monocyte sub-clusters in human peripheral blood sample via Uniform Manifold Approximation and Projection (UMAP) plots by cell identity (A) and different

stages (B). (C) A heatmap shows the top marker genes of monocyte sub-clusters. (D) Volcano plot illustrating the representative differential genes between post-treat group and pre-treat group of CD14+ monocyte (up) and IFITM2+ monocyte (down). (E) GO enrichment of upregulated (red) and downregulated (blue) DEGs of CD14+ monocyte (up) and IFITM2+ monocyte (down) in the post-treatment group compared to pre-treat group. (F) CD14+ monocyte (up) and IFITM2+ monocyte (down) subpopulation-specific regulons identified via SCENIC analysis. (G) Pseudotime trajectory (Monocle analysis) of the monocyte. Cells are colored based according to the predicted pseudotime (top) and groups(bottom). (H) The expression dynamics of top DEGs were cataloged into five major clusters in a pseudotime manner shown as red lines (cell fate 1) and blue lines (cell fate 2). (I) A heatmap shows the different expression patterns of top DEGs (cataloged in five clusters) along the development of Cell fate1 and cell fate 2. (J) GO enrichment analyses of each gene cluster.

Figure 4. Dynamic change process of T cell in response to JDM disease occurrence and treatment.

(A-B) Visualization of T cell sub-clusters in human peripheral blood sample via Uniform Manifold Approximation and Projection (UMAP) plots by cell identity (A) and different stages (B). (C) The feature plots of marker genes of T cell sub-clusters. (D) Heterogeneity of cell type composition of T cell sub-clustersin healthy control, pre-treatment, and post-treatment groups. The table shows the number of each cell type in each group (up); and the bubble chart represents the changes in the cell ratio of cell types between the pre-treatment and healthy control groups, and between the post- and pre-treatment groups in peripheral blood (down). (E) Pseudotime trajectory (Monocle analysis) of the CD4+ T cell sub-clusters. Cells are colored based according to the predicted pseudotime (left) and groups (right). (F) The expression dynamics of top DEGs were cataloged into five major clusters in a pseudotime manner. (G) A heatmap shows the different expression patterns of representative DEGs (cataloged in five clusters) along the reprogramming trajectory. Color key from blue to red indicates relative expression levels from low to high. (H) GO enrichment analyses of each gene cluster.

Figure 5. CD8+ T cells showed transition from a naive state to an inflammatory state and regulated by non-classical monocytes.

(A) Pseudotime trajectory (Monocle analysis) of the CD8+ T cell sub-clusters. Cells are colored based according to the predicted pseudotime (up) and groups (bottom). (B) The expression dynamics of top DEGs were cataloged into six major clusters in a pseudotime manner shown as red lines (cell fate 1) and blue lines (cell fate 2). (C) A heatmap shows the different expression patterns of of top DEGs (cataloged in six clusters) along the development of Cell fate1 and cell fate 2. (D) GO enrichment analyses of each gene cluster. (E-F) Selected ligand-receptor interactions (y-axis) against cell types (x-axis) from monocytes, CD4+ T cells (E) and CD8+ T cells (F) of human JMD samples. P values are indicated by circle size. The means of the average expression level of interacting molecule 1 in cluster 1 and interacting molecule 2 in cluster 2 are indicated by color.

Figure 6. Gene expression profile analysis of smooth muscle cells in JDM patients.

(A-B) Visualization of smooth muscle cell sub-clusters in human muscle sample via Uniform Manifold Approximation and Projection (UMAP) plots by cell identity (A) and different stages (B). (C) The feature plots of marker genes of smooth muscle cell sub-clusters. (D) GO enrichment analyses of marker genes of smooth muscle cell sub-clusters. (E) Pseudotime trajectory (Monocle analysis) of the smooth muscle cells. Cells are colored based according to the predicted pseudotime (top) and groups(bottom). (F) The expression dynamics of top DEGs were cataloged into six major clusters in a pseudotime manner. (G) Gene expression heatmap of top DEGs (cataloged in six clusters) in a pseudo-temporal order. (H) GO enrichment analyses of each gene cluster.

Figure 7. Fibroblasts with inflammatory gene expression profiles emerge in muscle samples of JDM patients .

(A-B) Visualization of fibroblast sub-clusters in human muscle sample via Uniform Manifold Approximation and Projection (UMAP) plots by cell identity (A) and different stages (B). (C) The feature plots of marker genes of fibroblast sub-clusters. (D) GO enrichment analyses of marker genes of fibroblast sub-clusters. (E) Pseudotime trajectory (Monocle analysis) of the fibroblast. Cells are colored based according to the predicted pseudotime (left) and groups (right). (F) The expression dynamics of top DEGs were cataloged into six major clusters in a pseudotime manner. (G) Gene expression heatmap of top DEGs (cataloged in six clusters) in a pseudo-temporal order. (H) GO enrichment analyses of each gene cluster.

Figure 8. Cellular interaction analysis between immune cells and smooth muscle cells or fibroblasts in JDM muscle samples.

(A-B) Bubble chart showing ligand-receptor relationship between immune cells and smooth muscle cells (A) or fibroblasts (B).
